# The PROMISE of Precision Medicine in Myocardial Infarction with Non-Obstructive Coronary Arteries

**DOI:** 10.3390/mps8030044

**Published:** 2025-04-27

**Authors:** Giulia La Vecchia, Vincenzo Scarica, Ludovica Leo, Rocco A. Montone

**Affiliations:** 1 Department of Cardiovascular and Pulmonary Sciences, Catholic University of the Sacred Heart, Largo Francesco Vito, 1, 00168 Rome, Italy; 2 Center of Excellence in Cardiovascular Sciences, Isola Tiberina Hospital Gemelli Isola, Via di Ponte Quattro Capi 39, 00186 Rome, Italy; 3 Department of Cardiovascular Sciences, Fondazione Policlinico Universitario A. Gemelli IRCCS, 00168 Rome, Italy

**Keywords:** MINOCA, precision medicine, targeted therapy

## Abstract

Myocardial infarction with non-obstructive coronary arteries (MINOCA) is a working diagnosis encompassing several pathophysiological mechanisms with specific treatments and different prognoses. Despite the absence of obstructive coronary artery disease, MINOCA has proven to be associated with a significant risk of mortality, angina burden, and socioeconomic costs. However, due to the heterogeneous nature of this clinical condition and the absence of randomized clinical trials, evidence supporting a standardized diagnostic algorithm and the clinical management of these patients is lacking. The PROMISE trial is the first randomized clinical trial evaluating the effectiveness of a precision medicine approach strategy in improving the outcomes and quality of life of patients with MINOCA, offering new insights into personalized treatment strategies. This review article discusses the promise of a precision medicine approach in patients with MINOCA, highlighting the potential innovations and challenges of a personalized medicine strategy in MINOCA.

## 1. Introduction

Myocardial infarction with non-obstructive coronary arteries (MINOCA) refers to different clinical entities presenting with the signs and symptoms of acute myocardial infarction (MI) in the absence of obstructive coronary artery disease (CAD) demonstrated at invasive coronary angiography (ICA) (no stenosis ≥ 50% in any major epicardial coronary artery) [1].

The term “MINOCA” was originally introduced in 2013 by Beltrame and colleagues, but it was only in 2017 that a position paper of the European Society of Cardiology (ESC) defined the diagnostic criteria of this clinical condition, which initially included both atherosclerotic and non-atherosclerotic causes of troponin raise [2,3]. In 2018, the fourth universal definition of MI underscored the difference between acute myocardial infarction and myocardial injury attributable to non-ischemic mechanisms [1]. Therefore, a position statement of experts in 2019 and the more recent ESC guidelines for the management of acute coronary syndromes (ACS) in 2023 reconceptualized the definition of MINOCA as referring to clinical scenarios due exclusively to an ischemic basis, excluding other non-ischemic causes of troponin elevations (e.g., myocarditis, Takotsubo Syndrome [TTS]) [4,5].

MINOCA is relatively common, with a prevalence varying from 3 to 15% across different studies [6,7]. Patients affected are usually younger, women, and with a lower prevalence of traditional cardiovascular risk factors [8]. Although it was initially believed to have a benign prognosis due to the absence of obstructive CAD, patients with MINOCA showed an increased risk of future cardiovascular (CV) events [9,10,11,12].

Furthermore, approximately 25% of patients with MINOCA will experience angina in the subsequent 12 months after the acute coronary event, thus portending a significant impact on quality of life and healthcare costs [11].

Despite the potential prognostic and therapeutic implications, there are few prospective clinical trials addressing this clinical condition and great variability still exists in the manner in which patients are evaluated and treated.

The “PROgnostic Value of Precision Medicine in Patients With Myocardial Infarction and Non-obStructive Coronary artEries” (PROMISE) study (ClinicalTrials.gov: NCT05122780) is a randomized, multicenter, prospective, open-label, superiority, and phase IV trial evaluating if a “precision-medicine approach”, including a comprehensive diagnostic work-up and a medical treatment specific to the underlying cause of MINOCA, will improve the quality of life and/or the prognosis of these patients as compared to the “standard” approach for the management of ACS [13]. The primary outcome will be the Seattle Angina Questionnaire (SAQ) score at 1-year of follow-up. Secondary outcomes will include MACEs (defined as a composite of all-cause mortality, re-hospitalization for MI, stroke, heart failure, and repeated ICA) at 1-year of follow-up, the healthcare costs and socioeconomic burden of a precision medicine vs. standard approach, and the additional value of cardiovascular magnetic resonance (CMR) and circulating biomarkers in further characterizing MINOCA. The efficacy of a precision medicine approach has been tested in patients with stable angina and/or signs of ischemia with no obstructive coronary artery disease (INOCA) in the coronary microvascular angina (CorMicA) trial, where a strategy of a full characterization of the specific endotype of INOCA and of a targeted medical therapy improved patients’ outcomes [14,15]. Evidence supporting the effectiveness of such an approach in the acute setting is lacking. The results derived from the PROMISE trial may potentially fill this knowledge gap and support a new pathophysiological-based approach in the management of patients with MINOCA.

## 2. Diagnostic Evaluation of MINOCA

**Figure 1:**
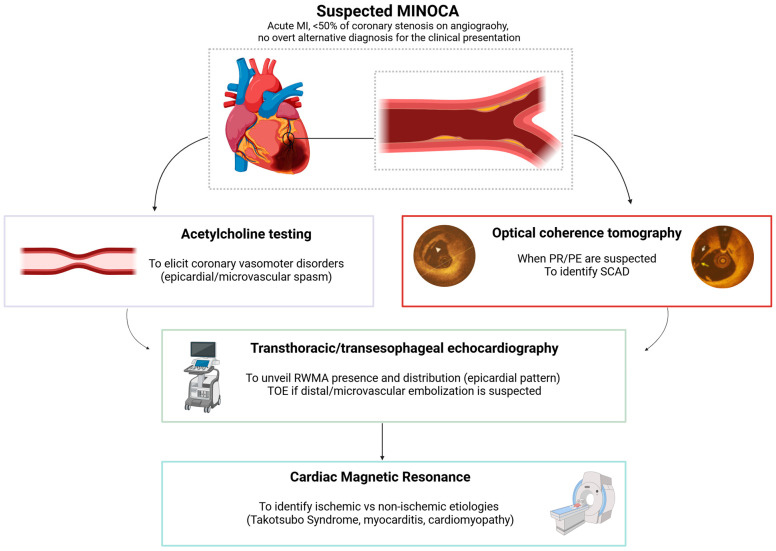
Diagnostic work-up of suspected MINOCA. The figure illustrates a comprehensive diagnostic work-up including both invasive (OCT and Ach provocative testing) and non-invasive (transthoracic and/or transesophageal echocardiography, cardiovascular magnetic resonance) tests aimed at identifying the specific etiology underlying MINOCA according to a precision medicine approach strategy. Abbreviations: MI: myocardial infarction; PE: plaque erosion; PR: plaque rupture; RWMA: regional wall motion abnormality; SCAD: spontaneous coronary artery dissection; TOE: transesophageal echocardiography. The figure has been created in Biorender. Montone, R.A. (2025) https://BioRender.com/.

In the clinical setting of an ACS with unobstructed CAD, MINOCA represents a working diagnosis. The management of these patients will vary depending on the underlying cause. Therefore, it is essential to follow a standardized, stepwise algorithm to establish the specific etiology of the acute clinical presentation (Figure 1) [16]. Firstly, the clinical context should be carefully reconsidered to rule out alternative, non-ischemic causes of acute myocardial injury (e.g., sepsis, pulmonary embolism, cardiac contusion, aortic dissection) [17]. Usually, patients presenting with ST-segment elevation ACS (STE-ACS) are transferred directly to the catheter lab as per the current standard of care pathway [5]. In this context, when non-obstructive coronary arteries are identified, a further invasive assessment should be considered, and non-invasive investigations can be performed once transferred to the ward or intensive care unit. On the other hand, in non-ST segment elevation ACS (NSTE-ACS) patients, the order in which the investigations are carried out may vary depending on the location where these subjects are managed during first medical contact [5].

In the PROMISE trial, patients with MI undergoing clinically indicated ICA are enrolled only after the MINOCA diagnosis has been confirmed by the absence of any epicardial coronary stenosis > 50% at ICA. MINOCA patients will then be randomized 1:1 using an online software available 24 h/24 h [13]. Individuals randomized in the “precision medicine approach” arm will undergo a comprehensive diagnostic work-up consisting of the following:

- ICA and ventriculography;
- An optical coherence tomography (OCT) study (if technically feasible);
- An intracoronary acetylcholine (Ach) provocative test (unless clinically contraindicated, i.e., due to hemodynamic instability, sustained ventricular arrhythmias);
- Transthoracic echocardiography (TTE) and CMR;
- Transesophageal echocardiography (TOE) (only if distal/microvascular embolization is suspected);
- Blood sampling for circulating biomarkers and microRNA (miRNA) expression profile.

On the other hand, patients randomized in the “standard approach” arm will undergo the following:

- ICA and left ventriculography;
- TTE;
- CMR if clinically indicated (e.g., when acute myocarditis or TTS are suspected).

### 2.1. Invasive Diagnostic Testing

Information regarding the exact pathogenic mechanism responsible for MINOCA, plaque vulnerability, or plaque burden cannot be obtained from coronary angiography alone [18,19,20,21]. Similarly, spontaneous coronary artery dissection (SCAD) or coronary functional abnormalities (epicardial or microvascular vasospasm) cannot be excluded from simple angiography [22]. Coronary imaging (including OCT and intravascular ultrasound [IVUS]) and invasive provocative tests overcome these limitations [19,23]. IVUS has long been used to characterize and quantify coronary plaque with an adequate depth, and to assist with percutaneous intervention. The ability of IVUS to identify the mechanism of acute MI was investigated among 50 women with MINOCA [24]. Of interest, in 38% of all cases, plaque disruption was identified as the pathogenetic mechanism of ACS, with 75% presenting with plaque rupture and 25% with plaque ulceration, despite the presence of mild CAD at coronary angiography [24]. OCT is an intravascular imaging technique that uses infrared light rather than ultrasound. The advantage of OCT is the 10-fold higher resolution compared to IVUS for inner wall structures and tissue characteristics, but it has reduced depth and coverage compared to IVUS [25,26]. In the presence of CAD, OCT can distinguish between stable coronary plaques (e.g., layered without thrombus) and the culprit lesion and allows for the further characterization of plaque disruption due to plaque rupture (PR), plaque erosion (PE), or erupted nodules [27]. The additional value of OCT in the diagnostic pathway of MINOCA was unveiled in the multicenter Women’s Heart Attack Research Program (HARP) study, including 145 MINOCA patients in whom OCT was performed during ICA [28]. The authors found that using OCT, a definite or possible culprit lesion could be identified in up to 46% of cases, unveiling a high prevalence of MINOCA due to atherosclerotic CAD [28]. A recent meta-analysis by Fedele and co-authors exploring the prevalence of different etiologies of MINOCA and their possible prognostic implications documented a prevalence of ACS due to plaque disruption of 47% among patients with a coronary stenosis of 1–49% when using OCT. These patients showed an increased 1-year risk of all-cause death or MI (RR = 1.49 [95% CI 1.17 to 1.90]) and MI alone (RR = 1.80 [95% CI 1.26 to 2.59]) compared to those presenting with undamaged coronary arteries, highlighting the potential clinical implications of further stratifying MINOCA [29]. The limitations of this OCT include costs, local availability, and expertise across different centers. Additional procedural times and contrast injections should also be considered, especially in patients with pre-existing renal disease [30]. In the PROMISE trial, patients randomized to the “precision medicine approach” undergo OCT when the ICA pattern suggests the presence of PR/PE (e.g., “haziness” appearance of the coronary) or SCAD (e.g., vessel or arterial wall contrast staining or gradual tapering of the vessel), and if not contraindicated due to technical reasons.

Coronary vasomotor disorders due to epicardial or microvascular spasms may be responsible for MINOCA in a significant proportion of patients, ranging from 3% to 95% of all cases [4,31]. Intracoronary provocative tests with ACh infusion can unveil these conditions by eliciting a vasoconstrictive response at both the epicardial and microvascular levels [32]. The test is considered to be positive for epicardial coronary vasospasms in the presence of epicardial coronary diameter reductions of ≥90% in comparison with the relaxed state which is associated with ischemic symptoms and electrocardiographic (ECG) changes [33]. Instead, microvascular angina is diagnosed in the presence of ischemic ECG abnormalities and angina but without epicardial vasospasm [33]. The ACh test proved to be safe in the acute setting with a low risk of transient complications, mainly represented by transient bradyarrhythmia and supraventricular tachycardia [34]. Moreover, it portended significant therapeutic and prognostic implications since MINOCA patients with a positive test were shown to present a higher prevalence of MACEs and major cerebrovascular events at follow-up [34].

Along with coronary angiography, cardiac ventriculography should be considered to identify areas of regional wall motion abnormalities (RWMAs) and wherever they match the territory supplied by a single epicardial coronary artery. If an epicardial pattern cannot be identified, non-ischemic etiologies may be responsible for the ACS, such as TTS, especially when an “apical ballooning” pattern (apical akinesia with basal walls hyperkinesia) is present [35].

### 2.2. Echocardiography

TTE does not have a primary role in identifying the pathogenetic mechanisms underlying MINOCA. However, it remains a cost-effective, non-invasive, and widely accessible tool, playing a key role in the initial evaluation of ACS by detecting RWMAs and ruling out alternative diagnoses [36].

TTE helps exclude MINOCA mimickers, including acute myocarditis, cardiomyopathy, and TTS [15]. The most common pattern of TTS is characterized by an apical hypokinesia/akinesia with basal wall hyperkinesia of the left ventricle (LV) identified at TTE and further confirmed by left ventriculography [37]. Less common patterns with RWMA localized to the mid-ventricular or basal regions and “focal” TTS are also described, thus making the differential diagnosis from ACS due to CAD and acute myocarditis challenging [38]. Echocardiographic findings in myocarditis are nonspecific. TTE may reveal the presence of pericardial effusion, wall thickening secondary to edema, and LV or biventricular dysfunction with RWMA not matching the territory supplied by a single epicardial coronary artery [38]. The diagnostic sensitivity of TTE may be enhanced through the assessment of ventricular strain via speckle-tracking echocardiography (STE), which has been demonstrated to correlate with late gadolinium enhancement (LGE) and the degree of inflammation on CMR [39,40]. However, for a definitive diagnosis, a multimodality imaging approach including CMR for tissue characterization should be pursued.

Finally, when a thromboembolic etiology is suspected as the underlying cause of MINOCA, contrast echocardiography and TOE contribute meaningfully to the diagnostic work-up through the detection of embolic foci, such as infective endocarditis, left ventricular apical thrombus, intracardiac tumors, prosthetic valves, or patent foramen ovale [41,42].

### 2.3. Cardiovascular Magnetic Resonance

CMR has emerged as a pivotal tool in the diagnosis and prognostication of MINOCA. It offers high-resolution imaging and tissue characterization that allows for the evaluation of myocardial edema, infarction, and fibrosis, distinguishing between ischemic and non-ischemic (e.g., myocarditis, cardiomyopathies, TTS) etiologies of suspected ACS and unobstructed coronary arteries [23,43,44,45,46]. In the PROMISE trial, all patients randomized to the “precision medicine approach” arm underwent CMR between day 3 and day 7 after the acute coronary event to underpin the specific cause underlying MINOCA [13]. On the contrary, those randomized in the “standard approach arm” underwent CMR during the index hospitalization if clinically indicated (e.g., due to the suspicion of acute myocarditis, cardiomyopathy, or TTS). It has to be considered that the trial was designed in 2019 when CMR had no clear recommendation for MINOCA patients [47,48]. More recently, the 2020 guidelines for the management of acute coronary syndromes in patients presenting without persistent ST-segment elevation stressed the importance of performing CMR in all patients with suspected MINOCA without an obvious underlying cause (class I level of evidence B) [49]. Further studies strengthened the role of CMR in reclassifying nearly 80% of MINOCA and in differentiating it from mimics [42,43,44]. A recent meta-analysis by Mileva et al. documented that among 26 studies including 3624 patients, CMR was able to reclassify the etiology of MINOCA in the majority of patients (68%) and that MI was confirmed as the final diagnosis only in a minority (22%) of cases [44]. Moreover, the ischemic etiology was associated with a 2.4-fold risk of major adverse cardiovascular events (OR: 2.40; 95% CI: 1.60–3.59), strengthening its therapeutic and prognostic implications for these patients. The ideal timeframe to perform CMR remains a cause of debate. The Stockholm Myocardial Infarction With Normal Coronaries 2 (SMINC) studies initially highlighted the valuable diagnostic yield of very early scanning (within 3 days from the initial clinical presentation), due to a higher possibility of detecting myocardial edema in the very acute phase of the disease, especially in those conditions with underlying transient causes (e.g., TTS) or limited myocardial injury (e.g., coronary vasospasm) [50,51]. However, performing a very early scan can be a relevant challenge in clinical practice due to the limited availability of this diagnostic tool across centers with different capabilities. More contemporary data demonstrated that even when performed within 14 days from presentation, CMR carries relevant diagnostic information with a likelihood of having a normal scan varying from 5% (with peak troponin of >211 ng/L) to 24% when myocardial injury is limited (peak troponin is <211 ng/L) [45]. These findings reinforce the role of CMR in the diagnostic pathway of patients with MINOCA.

## 3. Targeted Therapy Approach for MINOCA

The heterogeneous causes underlying the clinical presentation of MINOCA and the adverse clinical outcomes associated with this condition complicate the treatment approach of these patients.

Traditionally, MINOCA patients have been managed with the general secondary prevention strategies typically used for obstructive CAD, including dual antiplatelet therapy (DAT), statins, and beta-blockers [29]. In this regard, an observational study including 9136 patients from the SWEDEHEART registry (the Swedish Web-system for Enhancement and Development of Evidence-based Care in Heart disease Evaluated According to Recommended Therapy) assessed the effect of statins, DAT, and angiotensin-converting enzyme inhibitors (ACEi)/angiotensin receptor blockers (ARBs) on major adverse events (MACEs), including all-cause mortality, hospitalization for MI, ischemic stroke, and heart failure. The authors found that at a median follow-up of 4.1 years, statins and ACEi/ARBs significantly reduced the rate of MACEs (hazard ratios [HRs] of 0.77 [0.68–0.87] and 0.82 [0.73–0.93], respectively) [52]. Patients on beta blockers showed a trend toward a reduction in major adverse events (HR 0.86 [0.74–1.01]), while the effect of DAT was neutral (HR 0.90 [0.74–1.08]) [52]. A critical limitation of this study was the lack of definition of the specific causes of MINOCA. Therefore, this “standard of care” approach may not address the specific pathophysiological mechanisms underlying the clinical condition, which can span from ACS due to coronary plaque rupture, to vasomotor disorders of the coronary artery, micro embolism, etc. This is a critical point in the management of MINOCA. However, despite the recent evolution in the definition and diagnostic work-up, therapeutic management has not changed over the years, and, due to the limited evidence existing to date, current guidelines still do not address the issue of the acute and long-term management of these patients [5].

Against this background, the PROMISE trial seeks to shift the paradigm from the conventional treatment of obstructive CAD to a personalized approach tailored to the specific underlying causes of MINOCA, potentially improving the clinical outcomes of these patients.

According to the trial’s design, patients randomized to a precision medicine approach will receive a pharmacological and interventional treatment specific for the underlying cause etiology of MINOCA (Table 1). On the other hand, patients randomized to the standard approach arm will be treated with a standard secondary preventive strategy for ACS, including DAT or SAT and statins in all patients, beta blockers, and ACEi/ARBs, if clinically indicated [13].

There are a few other randomized clinical trials currently addressing this topic that will potentially shed light on the secondary prevention of patients with MINOCA. The “Stratified Medicine of Eplerenone in Acute MI/Injury” (StratMed-MINOCA) randomized clinical trial will assess if mineralocorticoid antagonist therapy (eplerenone) vs. standard of care therapy (without eplerenone) may limit myocardial damage in patients with MI/myocardial injury and coronary microvascular dysfunction (defined as an index of microvascular resistance [iMR] ≥ 25) (ClinicalTrials.gov: NCT05198791). The primary outcome will be a change in the biomarkers of myocardial damage (e.g., N-terminal prohormone of brain natriuretic peptide). Death and re-hospitalization rates will be evaluated as secondary clinical outcomes.

## 4. Discussion and Future Directions

The growing recognition of the prevalence and prognostic significance of MINOCA highlights the urgent need for precise diagnostic and therapeutic strategies. Despite advances in the acknowledgment of its pathophysiology, there is still significant variability in the diagnostic work-up and management of these patients. The PROMISE trial represents a crucial step toward a precision medicine approach, aiming to tailor treatments based on the specific underlying cause rather than applying a “one size fits all” strategy for unobstructive CAD. Future research should focus on integrating artificial intelligence and machine learning to develop new diagnostic algorithms for the early detection of the specific etiology and the identification of patients who may benefit from further invasive and non-invasive testing (e.g., CMR vs. OCT or IVUS) according to the clinical suspicion, ultimately improving resource use and reducing hospital stays.

Further fields of interest include identifying novel non-invasive biomarkers (e.g., circulating biomarkers and miRNAs) that can help in depicting the specific cause of MINOCA and assessing the long-term clinical outcomes of individualized therapies for these patients.

Gender-related differences across different etiologies are another unmet clinical need in the management of these patients. A recent observational study by Canton et al. highlights that among MINOCA patients, females ≤ 70 years old may represent an under-recognized high-risk group, with a higher incidence of major acute events [18 (23.7%) vs. 4 (5.9%); *p* = 0.003], mainly due to a higher re-hospitalization rate for HF (*p* = 0.045) and the recurrence of AMI (*p* = 0.006) [53]. These findings may be attributed to differences in the underlying pathophysiology of ACS between sexes, and to disparities in the management and the effectiveness of secondary prevention strategies across genders. These results underscore the urgent need for future sex-specific research in MINOCA, focusing on refining the diagnostic pathways and optimizing the treatment protocols for women affected by this condition.

## 5. Conclusions

The PROMISE trial is the first randomized clinical trial underscoring the potential advantages of a precision medicine approach in patients with MINOCA. As such, it represents a promising step forward in the care of these patients. By identifying the underlying cause of MINOCA, this approach could lead to more effective, targeted therapies and improve the quality of life for these patients. However, challenges related to the wide and heterogeneous nature of this condition, the need for specialized expertise, and the availability and cost of diagnostic tools remain significant barriers that need to be addressed. The results of the PROMISE trial could be pivotal in reshaping the clinical management of MINOCA.

## Figures and Tables

**Table 1:** Precision medicine approach therapies for different etiologies of MINOCA.

Underlying Mechanisms	Coronary Artery Plaque Disruption	Scad	Epicardial Coronary Artery Spasm	Microvascular Disorders	Coronary Embolism or Thrombosis
**DEFINITION**	PR: fibrous cap discontinuity with exposure of the plaque’s core to the bloodstream. PE: intact fibrous cap with an overlying thrombus and possible surface irregularities. Calcified Nodules: eruptive nodular calcification that protrudes into the arterial lumen.	Tear in the wall of an epicardial coronary artery which can obstruct coronary blood, caused by an intimal flap forming an intramural hematoma or vasa vasorum bleeding without endothelial rupture.	Chest pain, ST-segment changes, and vasoconstriction > 90% of an epicardial coronary artery in response to intracoronary provocative test with ACh or ergonovine.	Chest pain and ST-segment changes without vasoconstriction of an epicardial coronary artery in response to intracoronary provocative test with ACh or ergonovine.	Coronary artery obstruction due to embolic material or in situ thrombus.
**TAILORED THERAPEUTIC APPROACH**	- DAPT ± PCI - High-intensity statins - Beta blockers - ACEi/ARBs	- Conservative management in hemodynamically stable patients - PCI in high-risk cases (e.g., ongoing ischemia or hemodynamic instability) - Secondary prevention with SAPT, beta blockers, statin, and ACEi/ARBs	- CCBs - Nitrates (second choice) - Consider statins - Avoid beta-blockers due to risk of worsening vasospasm	- CCBs - Ranolazine (second choice)	- Treatment of the underlying cause - Anticoagulation

Abbreviations: ACEi: angiotensin-converting enzyme inhibitors; ACh: acetylcholine; ARBs: angiotensin receptor blockers; CCBs: calcium channel blockers; DAPT: dual antiplatelet therapy; PCI: percutaneous coronary intervention; PE: plaque erosion; PR: plaque rupture; SAPT: single antiplatelet therapy.

## Abbreviations

The following abbreviations are used in this manuscript:

ACEi	Angiotensin-converting enzyme inhibitors
Ach	Acetylcholine
ACS	Acute coronary syndrome
ARBs	Angiotensin receptor blockers
CAD	Coronary artery disease
CMR	Cardiac magnetic resonance
CV	Cardiovascular
DAT	Dual antiplatelet therapy
ECG	Electrocardiographic
ESC	European Society of Cardiology
ICA	Invasive coronary angiography
iMR	Index of microvascular resistance
IVUS	Intravascular ultrasound
MACE	Major adverse cardiovascular event
MI	Myocardial infarction
MINOCA	Myocardial infarction with non-obstructive coronary artery disease
OCT	Optical coherence tomography
PE	Plaque erosion
PR	Plaque rupture
RWMA	Regional wall motion abnormalities
SAQ	Seattle Angina Questionnaire
SCAD	Spontaneous coronary artery dissection
TOE	Transesophageal echocardiography
TTE	Transthoracic echocardiography
TTS	Takotsubo Syndrome
